# Deficiencies of proteins C, S and Antithrombin and factor V Leiden and the risk of ischemic strokes

**Published:** 2010-08-25

**Authors:** AM Soare, C Popa

**Affiliations:** Neurology Clinic, National Institute of Neurology and Neurovascular Diseases, BucharestRomania; ** Medical University in PoznanPoland

**Keywords:** thrombophilia, coagulopathy, stroke, patent foramen ovale

## Abstract

Although hypercoagulable states are most often associated with venous thromboses, arterial thromboses are reported in protein C, protein S, antithrombin deficient patients and in those with factor V Leiden, components of hereditary thrombophilia. Because these arterial thromboses (peripheral artery disease, myocardial infarction, and cerebral infarction) mostly affect young persons, aged below 45 years, it is important to test and treat these thrombophilic defects. Because the relation thrombophilia – arterial thromboses is still under debate, due to conflicting data, this article is a review of studies published in literature regarding the implication of the above–mentioned thrombophilic defects in cerebral infarcts.

## Introduction

Most patients with arterial stroke have diabetes, hypertension, hyperlipidemia, smoking or valvular heart disease as underlying risk factors. When such risk factors are absent, one should suspect hypercoagulable states as causative. The inherited hypercoagulable syndromes primarily affect veins and only rarely cause arterial thrombosis. The predilection to venous as opposed to arterial stroke may be partly due to the differing mechanisms of thrombosis on the venous and arterial sides of the circulation and within various organs. Stasis is a major predisposing factor in venous circulation whereas endothelial damage is a more important predisposing factor in arterial circulation. Because there are still conflicting data regarding the implications of protein C, protein S, antithrombin deficiencies and factor V Leiden mutation in arterial stroke, the present article is a review of the published studies debating this issue. 

Regarding the cerebral infarctions, the implications of protein S, C, and antithrombin deficiencies is still on debate; more certain is the implication of factor V Leiden. 

Half of the ischemic strokes are unexplained by conventional risk factors and the genetic predisposition has been deemed responsible for some of these risks. (Table 1) shows the monogenic causes of ischemic stroke.  

**Table 1:** Monogenic causes for ischemic stroke [1]

Disorders	Gene/chromosomal location responsible
Small vessel disease	
CADASIL	notch 3 gene
CARASIL	Unknown
Cerebroretinal vasculopathy and HERNS	3p21.1–21.3
Large artery disease	
Dyslipidaemias	Various
Moyamoya disease	3p24.2–26 and 17q25
Pseudoxanthoma elasticum	ABCC6 gene
Neurofibromatosis type 1	NFI gene
Disorders affecting both small and large arteries	
Fabry disease	alpha–galactosidase A gene
Homocystinuria	cystathione beta synthase gene
Sickle cell disease	Methylene tetrahydrofolate reductase
Cardioembolic	
Cardiomyopathies: primary/secondary	Various
Familial dysrythmias	Various
Prothrombotic disorders	
Protein C, S deficiency	Protein C gene – 2q13–q14 chromosome, Protein S gene –3p11.1–3q11.2 chromosome
Antithrombin 3 deficiency	Antithrombin 3 gene– 1q23–25 chromosome
Familial anticardiolipin syndrome	Unknown
Activated protein C resistance	Factor V Leiden mutation G1691A mutation in factor V gene – 1q21–25 chromosome
Arterial dissection	
Ehlers–Danlos syndrome type 4	collagen type 3 gene
Fibromuscular dysplasia	Unknown
Marfan syndrome	fibrillin–1 gene
Mitochondrial disorders	
MELAS	Mitochondrial DNA mutations

CADASIL, cerebral autosomal dominant arteriopathy with subcortical infarcts and leucoencephalopathy; CARASIL, cerebral autosomal recessive arteriopathy with subcortical infarcts and leucoencephalopathy; HERNS, hereditary endotheliopathy with retinopathy, nephropathy, and stroke; MELAS; mitochondrial myopathy, encephalopathy, lactic acidosis, and stroke like episodes; 

Stroke means different pathological processes, all of them having as an end point the focal cerebral ischemia. 85% of the strokes are ischemic and 15% hemorrhagic. Even among the patients with ischemic stroke, a number of pathologically different processes are responsible, as cardioembolism, large artery disease with arteriolosclerosis and thromboembolism, and small artery disease. The physiopathological mechanisms and the genetic influences responsible could be different for different stroke subtypes. Two family studies showed an association with large artery and small artery strokes, but not with cardioembolic stroke [2,3]. The association was stronger in cases of stroke in young age. In one study regarding the role of factor V Leiden mutation and ischemic stroke subtypes, it was proven that it is statistically significant more frequently in patients with large infarctions (13.6%; p < 0.025; OR 2.25, CI 1.16–4.34) than in those without stroke (6.5%), therefore this mutation may predispose the person to large cerebral infarction. It has not been proved that it is a risk factor for ischemic stroke in general [4].

The prevalence of protein C, S and antithrombin 3 in ischemic stroke varies up to 23% in different studies [5].   

In an ample meta–analysis published in 2003, reports of cases and studies regarding the deficiencies of proteins S, C, antithrombin, and factor V Leiden in ischemic strokes were mentioned [6]. Protein C deficiency has occasionally been associated with arterial ischemic stroke [7]. The meta–analysis mentions reports of cases and studies that had a frequency of 4%, 5%, 6%, 11% and even 21% in the groups of stroke patients, depending on the admission criteria. These patients had strokes at a younger age than those without protein C deficiency. One study in which only one of the 329 patients aged between 15 and 45 years had protein C deficiency is mentioned. The conclusion of the meta–analysis was that it appears that protein C deficiency is weakly associated with the arterial stroke [6]. 

Protein S deficiency has been associated with cerebral arterial ischemia more often than has protein C deficiency. However, conflicting reports limit the reliability of this association. The meta-analysis describes case–reports and studies published until 2003 regarding this subject: they reported a frequency of this deficiency of 13,8% (5/36), 19% (8/35), 23% (19/98) in patients aged less than 45 years and  6% (4/66) in patients aged less than 60 years [6]. In this last study, all protein S deficiency patients had elevated anticardiolipin antibodies. This study suggests an association between antiphospholipidic syndrome and protein S deficiency in ischemic stroke patients. Conversely, numerous studies show a less frequent association of stroke and protein S deficiency (Table 2). In the Iowa cohort study, only 1 of the 329 stroke patients aged between 15 and 45 years had protein S deficiency. A Swedish study of 107 patients aged between 18 and 44 years found only one with protein S deficiency [6] . 

**Table 2:** Coagulopathies and arterial stroke[6]

Coagulopathy	Association with arterial stroke
Protein C deficiency	weak
Protein S deficiency	moderate
Antithrombin 3 deficiency	rare
Factor V Leiden mutation	moderate
Prothrombin gene mutation	moderate
Hyperhomocysteinemia	moderate
Dysfibrinogenemia	rare
Plasminogen deficiency	rare
Sickle cell anemia	common
Antiphospholipid antibodies	common

Antithrombin deficiency has only rarely been associated with stroke. The meta–analysis presents case–reports and studies regarding the association of this deficiency with stroke. Different studies show frequencies of 5% (3/66), 8% (5/60), but there were studies that reported just one case of the 36 or 329 cases studied. It is worth mentioning that all studies included patients aged less than 45–55 years, and the study that reported a frequency of 5%, all antithrombin deficiency patients had suffered carotid artery territory strokes [6]. Thus, the evidence linking antithrombin deficiency with arterial stroke is weak.

The factor V Leiden mutation, the cause of activated protein C resistance (in 90% of cases) is the most common inherited coagulopathy associated with stroke. The meta–analysis reports a prevalence of the mutation of 10% (3/30), 12,3%(10/81), according to different studies and 2 studies that included stroke patients aged younger than 45 years with cryptogenic cerebral ischemia found a prevalence of 12% and 15,9%, respectively[6].

In 2004, a meta–analysis of all the studies made until January 2003, was published in English–language journals and it was related to the investigation of any candidate gene for ischemic stroke in humans. In order to maintain genetic homogeneity, only studies in white adults were included. Data from 120 case–control studies were included. Pooled odds ratios with 95% confidence intervals (CIs) were included. Of the 32 genes studied, 15 polymorphisms were identified. Statistically significant associations with ischemic stroke were identified only for factor V Leiden Arg506Gln (OR, 1.33; 95% CI, 1.12–1.58), but not for the other 3 deficits [8].

A study performed in Turkey and published in 2005, studied 29 children with ischemic stroke and 20 with intracerebral hemorrhage, all of whom were compared with 20 controls. The authors found no evidence of an association between factor V Leiden mutation and ischemic stroke or intracerebral hemorrhage. The conclusion was that factor V Leiden mutation did not seem to be associated with a risk of cerebrovascular disease [9]. Another study performed in the same country, published in 2007 had retrospectively assessed the risk factors of children with arterial stroke. It was proven that among the 31 patients, 2 had factor V Leiden, 2 protein C deficit, 2 antithrombin deficit and one protein S deficit. This study concluded that for the correct etiologic identification, prothrombotic risk factors should be extensively evaluated in patients with arterial ischemic stroke [10]. A study performed in Italy and published in 2008, regarding peri–neonatal ischemic stroke in a group of 24 children, found inherited thrombophilia (factor V Leiden mutation, prothrombin gene mutation and proteins C, S and antithrombin deficiencies) in 28,6% cases. Inherited thrombophilia was significantly more prevalent in patients with bad neurologic outcome (Fisher's exact test P = 0.002) [11]. 

The risk of a stroke is high for persons with factor V Leiden combined with other vascular risk factors, such as smoking and contraceptive use. A study investigated the prevalence of these mutations in 468 patients with an acute stroke or transient ischemic attack (TIA) before the age of 60 and in a healthy control population individually matched for age and gender. A significant interaction between the factor V Leiden, smoking, and risk of stroke in women was found: female smokers without the factor V Leiden had a somewhat increased risk of stroke of 2.6 (95% CI, 1.5 to 4.6; P=0.001) compared with nonsmoking non–carriers of the factor V Leiden. No such interaction was observed in men [12].

Nowadays, there are many studies about the association between patent foramen ovale and thrombophilic defects that lead to a high incidence of ischemic strokes, especially in young persons.

Patent foramen ovale (PFO) is present in  25% of the general population, but in most cases, it does not affect health. But, this anomaly represents a possible cause of ischemic embolic neurological events in young patients [13,14]. The relation between patent foramen ovale and cryptogenic thromboembolic cerebral events has not been established and the suggested mechanisms are complex, including the paradoxical embolism from the periferic venous system, embolisms from thrombs formed in atrial septum and because of the transitory atrial arrhythmias [15]. It is believed that prothrombotic mutations are genetic risk factors for cryptogenic ischemic cerebrovascular events in young subjects with patent foramen ovale and there is an association between young patients with PFO and the risk of cerebral ischemia [16,17,18].

One study that included 97 young patients with PFO and 160 age–matched control subjects found that the combination of either factor V Leiden or prothrombin G20210A and PFO was associated with a 4.7–fold (95% CI=1.4 to 16.1; P=0.008) increased risk of cerebral ischemia in young patients. No statistically significant association was found for protein C, S or antithrombin deficiency [19]. Of the inherited thrombophilias, factor V Leiden and the prothrombin 20210 mutation have been associated with stroke, but this association is statistically significant only in children and adults under the age of 40. The risk of stroke in persons with these mutations is substantially increased by concomitant exposure to oral contraceptives [20]. In another study published in 2009 in Thrombosis and Haemostasis, that analyzed data from six eligible studies regarding the association of factor V Leiden with PFO, a trend toward an association for the FV(G1691A) mutation (OR 1.18; 95% CI 0.73 to 1.90, compared to control subjects was noticed; OR 1.14; 95% CI 0.62 to 2.09, compared to non–PFO–associated stroke patients). The status of carrier of FV(G1691A) mutation was associated with a risk for stroke of 1.98 (95% CI 1.38 to 2.83) and 1.62 (95% CI 1.03 to 2.57), as compared to control subjects and non–PFO–associated stroke patients, respectively. Additional common prothrombotic genetic variants to standard initial screening may contribute to ranging PFO-associated stroke patients according to a different risk of ischemic events and targeting secondary prevention strategies [21]. 

It was presumed that the presence of inherited thrombophilic defects could induce the potential risks and reduce the benefits of percutaneous closure of patent foramen ovale [22]. A study that investigated 72 patients with recurrent brain ischemia concluded that for cryptogenic stroke patients the association between PFO and thrombophilia significantly raises the risk for recurrences and percutaneous closure of patent foramen ovale is efficient in order to prevent recurrences in patients with thrombophilia [22].

## Conclusions

Dealing with a case of cerebral infarction with few risk factors for arteriosclerosis, the screening for thrombophilia should be made, especially when patients are aged below 45 years, have a personal history of recurrent thrombosis without precipitating factors, thrombosis in unusual sites, thrombosis during pregnancy, a positive family history of thrombosis. A positive result asks for initiation of the adequate anticoagulant treatment.
